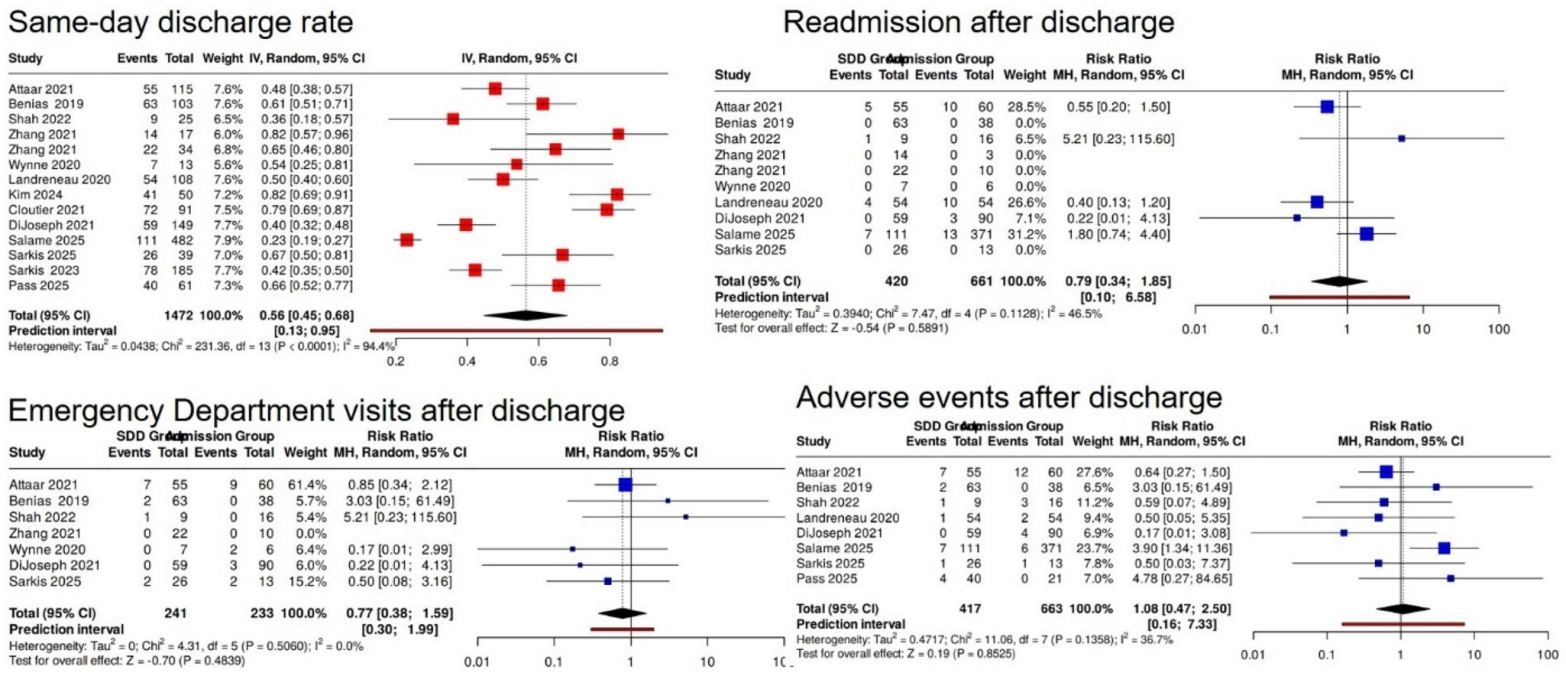# Poster Session I - A108 SAME-DAY DISCHARGE AFTER THIRD-SPACE ENDOSCOPY: A SYSTEMATIC REVIEW AND META-ANALYSIS

**DOI:** 10.1093/jcag/gwaf042.108

**Published:** 2026-02-13

**Authors:** M Fujiyoshi, N Sayed, M Monaghan, D Tham, Y Fujiyoshi

**Affiliations:** Division of Gastroenterology, University of Ottawa, Ottawa, ON, Canada; Division of Gastroenterology, University of Ottawa, Ottawa, ON, Canada; Division of Gastroenterology, University of Ottawa, Ottawa, ON, Canada; Division of Gastroenterology, University of Ottawa, Ottawa, ON, Canada; Division of Gastroenterology, University of Ottawa, Ottawa, ON, Canada

## Abstract

**Background:**

Third-space endoscopic procedures, including peroral endoscopic myotomy (POEM) for achalasia, Zenker’s POEM (Z-POEM) for Zenker’s diverticulum, and gastric POEM (G-POEM) for gastroparesis, have traditionally involved several days of short inpatient observation. Emerging reports suggest same-day discharge (SDD) may be feasible and safe.

**Aims:**

To evaluate the feasibility and safety of SDD after third-space endoscopy (POEM, Z-POEM, and G-POEM).

**Methods:**

We systematically searched MEDLINE, Embase, the Cochrane Central Register of Controlled Trials (CENTRAL), and grey literature from inception to March 2025. Studies reporting SDD after third-space endoscopy were eligible. The primary outcome was the SDD rate. Secondary outcomes were 30-day readmission, emergency department (ED) visits, and post-discharge adverse events (AEs). We pooled proportions and calculated risk ratios (RRs) with 95% confidence intervals (CI) comparing SDD with admission. Random-effects models were used and heterogeneity was quantified using I^2^.

**Results:**

Of 175 records identified, 31 duplicates were removed, and 142 titles/abstracts were screened; 14 studies met inclusion criteria. The pooled SDD rate was 56% (95% CI, 45–68, I^2^= 94.4%). Compared with admission group, SDD was not associated with higher readmission (RR, 0.79; 95% CI, 0.10–6.58, P = 0.59, I^2^= 46.5%), ED visits (RR, 0.77; 95% CI, 0.38–1.59, P = 0.48, I^2^ = 0.0%), or post-discharge AEs (RR, 1.08; 95% CI, 0.47–2.50, P = 0.85, I^2^ = 36.7%).

**Conclusions:**

Across 14 studies, over half of patients underwent SDD after third-space endoscopy without increased readmissions, ED visits, or post-discharge AEs versus admission. These findings support the feasibility and safety of SDD for appropriately selected patients. Prospective studies using standardized SDD criteria and procedure-specific protocols are warranted.

**Funding Agencies:**

None